# Computer-aided diagnosis of keratoconus through VAE-augmented images using deep learning

**DOI:** 10.1038/s41598-023-46903-5

**Published:** 2023-11-23

**Authors:** Zhila Agharezaei, Reza Firouzi, Samira Hassanzadeh, Siamak Zarei-Ghanavati, Kambiz Bahaadinbeigy, Amin Golabpour, Reyhaneh Akbarzadeh, Laleh Agharezaei, Mohamad Amin Bakhshali, Mohammad Reza Sedaghat, Saeid Eslami

**Affiliations:** 1 Pharmaceutical Research Center, Pharmaceutical Technology Institute, Mashhad University of Medical Sciences, Mashhad, Iran; 2 Department of Medical Informatics, Faculty of Medicine, Mashhad University of Medical Sciences, Mashhad, Iran; 3 Department of Computer Engineering, Ferdowsi University of Mashhad, Mashhad, Iran; 4 School of Paramedical Sciences and Rehabilitation, Mashhad University of Medical Sciences, Mashhad, Iran; 5 Eye Research Center, Mashhad University of Medical Sciences, Mashhad, Iran; 6 Medical Informatics Research Center, Institute for Future Studies in Health, Kerman University of Medical Sciences, Kerman, Iran; 7 School of Medicine, Shahroud University of Medical Sciences, Shahroud, Iran; 8 Department of Optometry, School of Paramedical Sciences, Mashhad University of Medical Sciences, Mashhad, Iran; 9 Modeling in Health Research Center, Institute for Future Studies in Health, Kerman University of Medical Sciences, Kerman, Iran

**Keywords:** Data mining, Medical imaging

## Abstract

Detecting clinical keratoconus (KCN) poses a challenging and time-consuming task. During the diagnostic process, ophthalmologists are required to review demographic and clinical ophthalmic examinations in order to make an accurate diagnosis. This study aims to develop and evaluate the accuracy of deep convolutional neural network (CNN) models for the detection of keratoconus (KCN) using corneal topographic maps. We retrospectively collected 1758 corneal images (978 normal and 780 keratoconus) from 1010 subjects of the KCN group with clinically evident keratoconus and the normal group with regular astigmatism. To expand the dataset, we developed a model using Variational Auto Encoder (VAE) to generate and augment images, resulting in a dataset of 4000 samples. Four deep learning models were used to extract and identify deep corneal features of original and synthesized images. We demonstrated that the utilization of synthesized images during training process increased classification performance. The overall average accuracy of the deep learning models ranged from 99% for VGG16 to 95% for EfficientNet-B0. All CNN models exhibited sensitivity and specificity above 0.94, with the VGG16 model achieving an AUC of 0.99. The customized CNN model achieved satisfactory results with an accuracy and AUC of 0.97 at a much faster processing speed compared to other models. In conclusion, the DL models showed high accuracy in screening for keratoconus based on corneal topography images. This is a development toward the potential clinical implementation of a more enhanced computer-aided diagnosis (CAD) system for KCN detection, which would aid ophthalmologists in validating the clinical decision and carrying out prompt and precise KCN treatment.

## Introduction

Keratoconus (KCN) is a non-inflammatory disease characterized by bilateral progressive corneal thinning that results in an abnormally steep cornea, decreased vision, and potentially leading to vision loss if it is not detected and treated at an early stage^1^. Although the underlying causes of KCN are unknown, Ophthalmologists, link it to chronic disease, eye rubbing, and genetic inheritance. KCN progression may proceed quickly or slowly, and it may come to an end at some point^2^. Keratoconus affects 1 in 2000 people in the general population, and both the prevalence of the condition in adults and children has considerably increased in recent years^3^. Asian populations have approximately four times higher prevalence of KCN compared to other ethnic groups^4^, with the highest prevalence observed in the Mediterranean region and the Middle East, such as Iran^5^. According to epidemiological studies released in recent years, just 0.3 cases per 100,000 persons 0.0003% are reported in Russia, 2.3% in India, and 2.5% in Iran^6^.

In the field of machine learning, researchers face a significant challenge in obtaining sufficient medical image datasets. This is due to the difficulty in capturing such data, as well as the time-consuming process of acquiring and labeling it, which requires considerable effort from both researchers and specialist^7^. To address the issue of limited datasets, various studies have explored the use of data augmentation, a popular technique in computer vision^8^. AI has advantages over human evaluation in terms of data processing, information integration, and diagnostic speed. To date, many methods for implementing machine learning, such as support vector machines, decision trees, or neural networks, have been recommended. In many scientific fields, multilayered neural networks, specifically convolutional neural networks (CNNs), have recently accomplished outstanding effects in a variety of image classifications^9^. Several studies have employed machine learning to identify keratoconus^10–14^, however the majority have either used topographic numeric indices obtained with a Placido disc-based corneal topographer or tomographic numeric indices acquired using a scanning slit tomographer and a rotating Scheimpflug camera. The impressive capabilities of convolutional neural networks (CNNs) in pattern recognition and image classification tasks make them a highly suitable option for automating the analysis of color-coded images^15–18^. Deep learning methods, in particular deep convolutional neural networks (CNNs), have been used to identify KCN using color-coded corneal maps of elevation, curvature, and thickness^19–23^. Despite the fact that DL models generally need a larger number of samples, these research typically used limited subsets of images with fewer than 400 images^20^. Zeboulon et al.^22^ detected KCN and a history of refractive surgery using a sizable dataset with 3000 corneal images. They distinguished KCN from normal with a high degree of accuracy. Additionally, since developing and refining deep CNN models is often computationally expensive, models that execute more quickly, like our current model, have a better chance of being incorporated in clinical settings.

In this study, an innovative approach involving variational auto encoders (VAE) was employed to generate and augment images. we employed a substantial dataset comprising 4000 corneal images to train and assess four deep convolutional neural network (CNN) models for the purpose of diagnosing keratoconus. Three of these methods utilized transfer learning and fine-tuning of pretrained models on a customized dataset. The fourth method employed a customized CNN as a proposed model developed from scratch. Each model became an expert at identifying KCN features from that specific corneal map and a variety of topographic patterns, instead of complex topographic indexes.

## Results

The keratoconus group consisted of 475 patients, 275 men and 200 women, with the mean age of 33.27 ± 8.09 years. The normal group consisted of 535 subjects who were refractive surgery candidates, had 188 men and 347 women, with the mean age of 34.56 ± 8.76 years. The keratoconus group was younger than the normal group and substantially different were noted concerning sex distribution (Table 1).

**Table 1:** Characteristics of population.

Attributes	Keratoconus (n = 475)	Normal (n = 535)	P value
	Mean ± SD	Mean ± SD	
Age (years)	33.27 ± 8.09	34.56 ± 8.76	0.03
Frequency (%)			
Sex			
Male	275 (57.9)	188 (35.1)	< 0.001
Female	200 (42.1)	347 (64.9)	

Table 2 presents the descriptive statistics of topographical parameters for both groups, indicating significant differences in all indices between the keratoconus and normal groups.

**Table 2:** Topographic parameters of the keratoconus and the normal.

Attributes	Keratoconus (n = 475)	Normal (n = 535)	P value
	Mean ± SD	Mean ± SD	
AveK (D)			
OD	48.66 ± 3.92	44.11 ± 1.66	< 0.001
OS	48.7 ± 4.00	44.18 ± 1.49	< 0.001
Total	48.68 ± 3.96	44.15 ± 1.57	< 0.001
Cyl (D)			
OD	4.77 ± 2.48	1.57 ± 1.50	< 0.001
OS	4.52 ± 2.58	1.65 ± 1.46	< 0.001
Total	4.64 ± 2.53	1.61 ± 1.48	< 0.001
SRI (D)			
OD	0.83 ± 0.45	0.16 ± 0.23	< 0.001
OS	0.82 ± 0.43	0.17 ± 0.21	< 0.001
Total	0.82 ± 0.44	0.17 ± 0.22	< 0.001
SAI (D)			
OD	1.92 ± 1.07	0.37 ± 0.21	< 0.001
OS	1.93 ± 1.09	0.39 ± 0.20	< 0.001
Total	1.93 ± 1.07	0.38 ± 0.20	< 0.001

*AveK* average keratometry, *D* diopter, *Cyl* cylinder, *SRI* surface regularity index, *SAI* surface asymmetric index.

**Figure 1:**
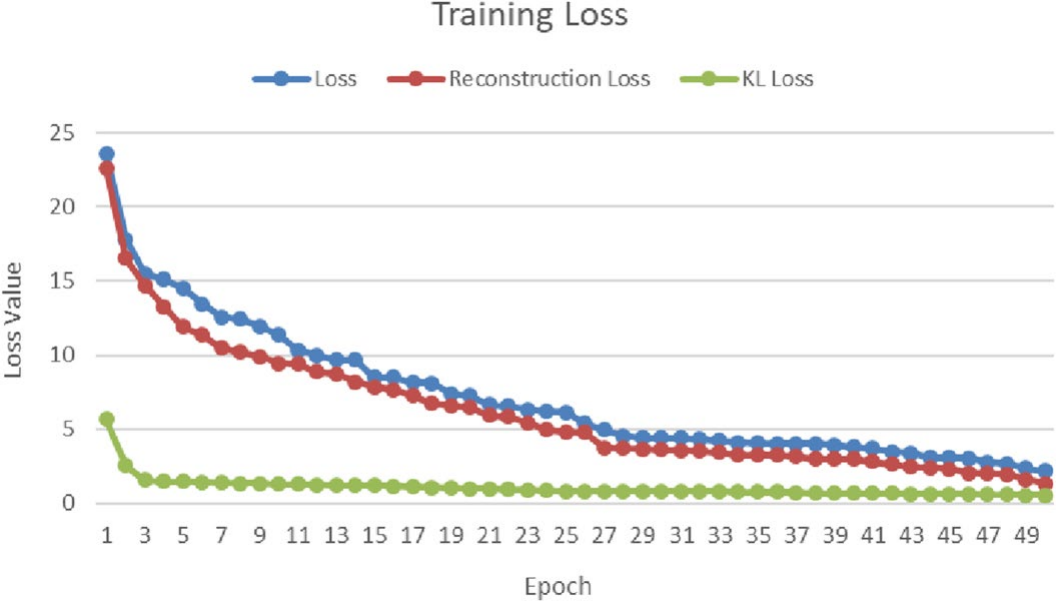
VAE model training loss metrics.

In order to address the challenge of limited data for deep learning training and to expand our dataset, we conducted training using VAE generative models. Our training encompassed the entire dataset, which comprised a total of 1748 images. This dataset was divided into two categories: 978 images categorized as ‘Normal’, and 780 images categorized as ‘Keratoconus’. The training process resulted in a cumulative loss of approximately 6.667. This loss included a reconstruction loss of 5.533 and a Kullback–Leibler loss of approximately 1.133 for the latest iteration of our models (Fig. 1).

**Figure 2:**
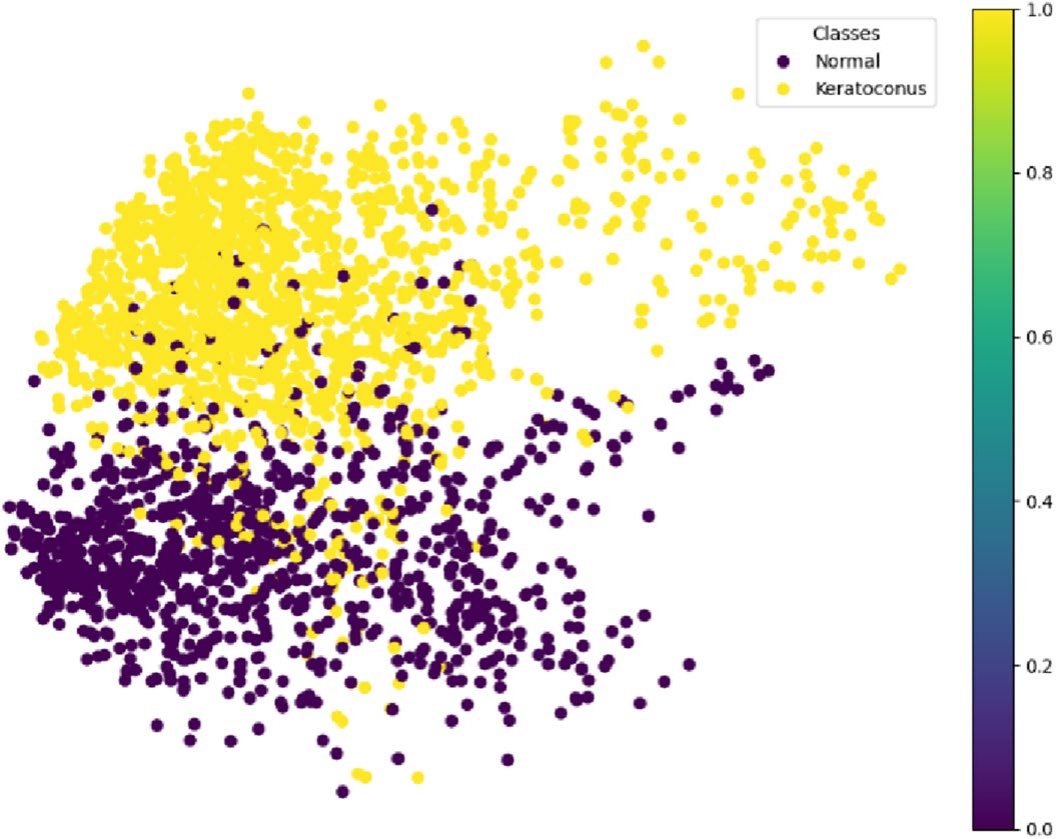
Visualization of clustering by VAE.

We opted to train our generative models on the entire dataset, encompassing images from both classes. This decision was based on the efficiency demonstrated by VAE networks in clustering and discerning feature distinctions between these classes. This efficiency is particularly evident when VAE functions as an unsupervised model solely aimed at uncovering data patterns. Notably, our experimentation has confirmed that training a generative model like VAE on the Normal and Keratoconus classes separately yields no discernible advantage over training on both image types simultaneously, as clearly depicted in Fig. 2. This underscores the significant pattern recognition capabilities of these models.

**Figure 3:**
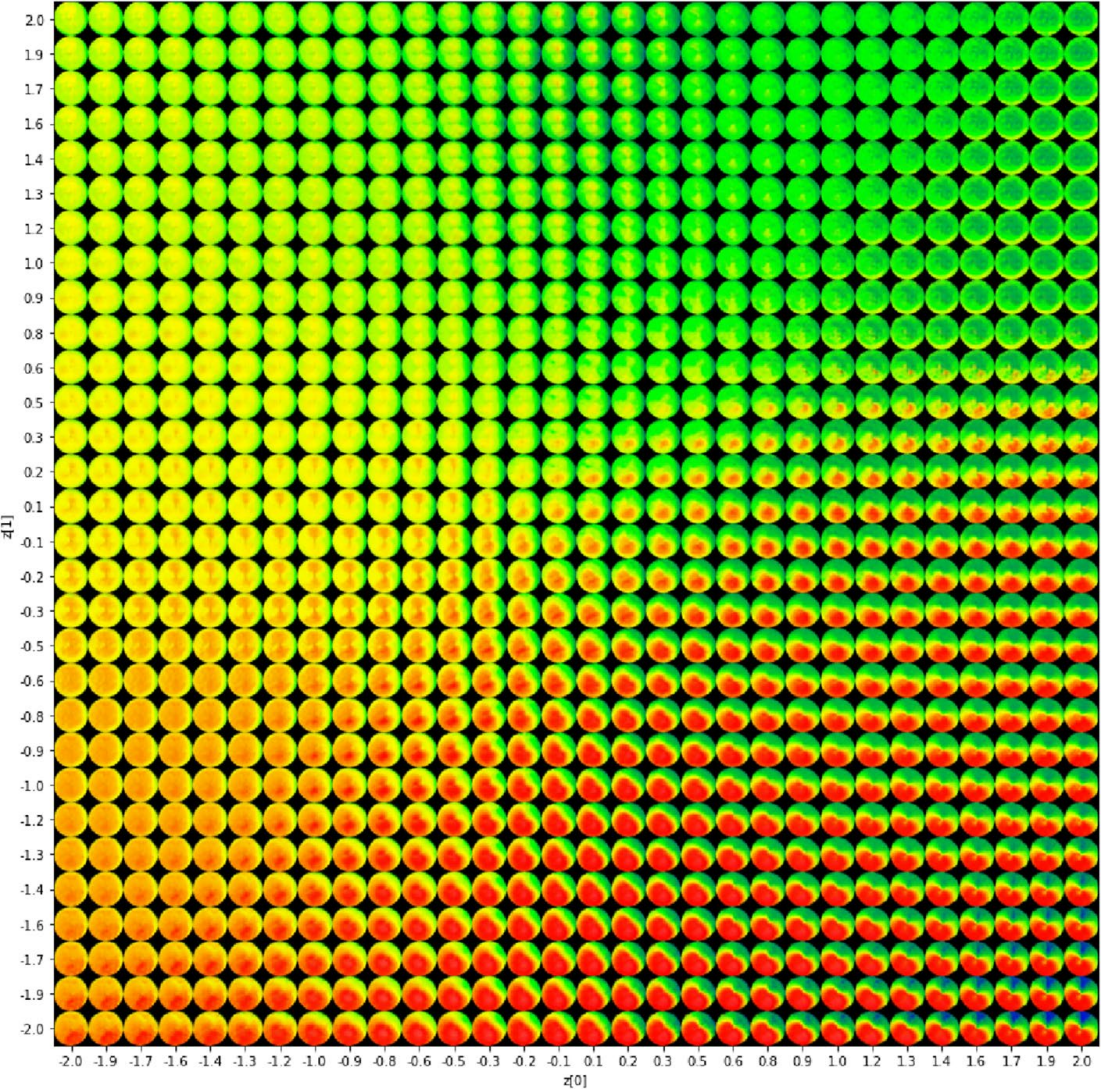
900 outputs of reconstruction process varying among discriminative cone types.

For improved visualization of our network's outcomes, we adopted a method wherein we designated a specific range of mean (μ) and standard deviation (σ) parameters within the latent space. These parameters were meticulously selected to generate diverse sets of latent variables (Z), which were then employed as inputs for our pre-trained decoder model. In our study, the decoder model's weights, having undergone careful training on our dataset, were loaded and initialized with these varied latent variable inputs. This approach allowed us to generate a series of images across the specified parameter range. Specifically, we employed 30 different values for both mean and standard deviation parameters, maintaining a consistent step and separation between each value. As a result, we created a total of 900 novel image samples, encompassing various types and patterns, as depicted in Fig. 3.

**Figure 4:**
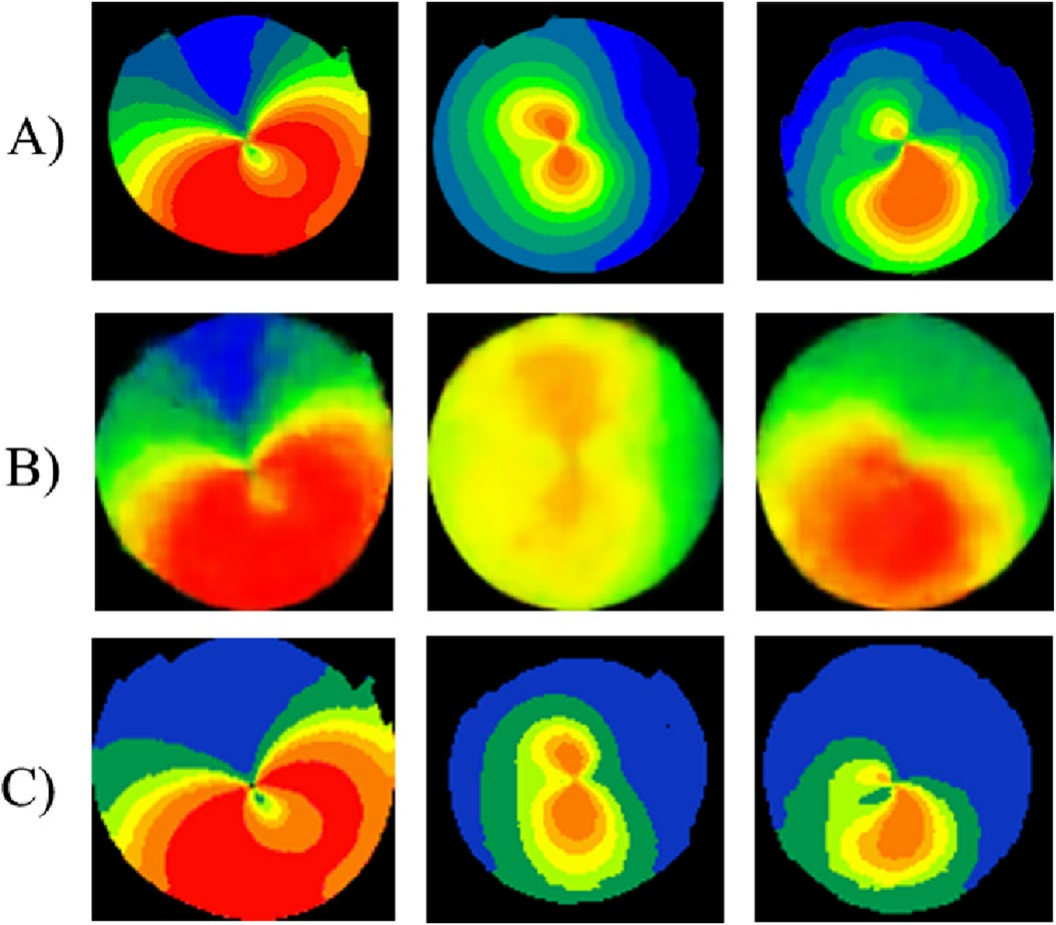
An overview of progression in our VAE outputs through developing various versions. Original images (**A**). Initial model outputs (**B**). Final generated images (**C**).

As demonstrated in Fig. 4, (A) illustrates the original images utilized as a part of test dataset. (B) demonstrates the initial version of outputs by training VAE model using data from only one clinic. Subsequently, through an increase in the number of images from multiple clinics and Optimizing VAE model, the final version of outputs achieved a satisfactory level of quality and confidence in learning significant discriminative cone types patterns (C). According to Fig. 4, the model has the ability to produce synthetic images that closely resemble the original images.

**Figure 5:**
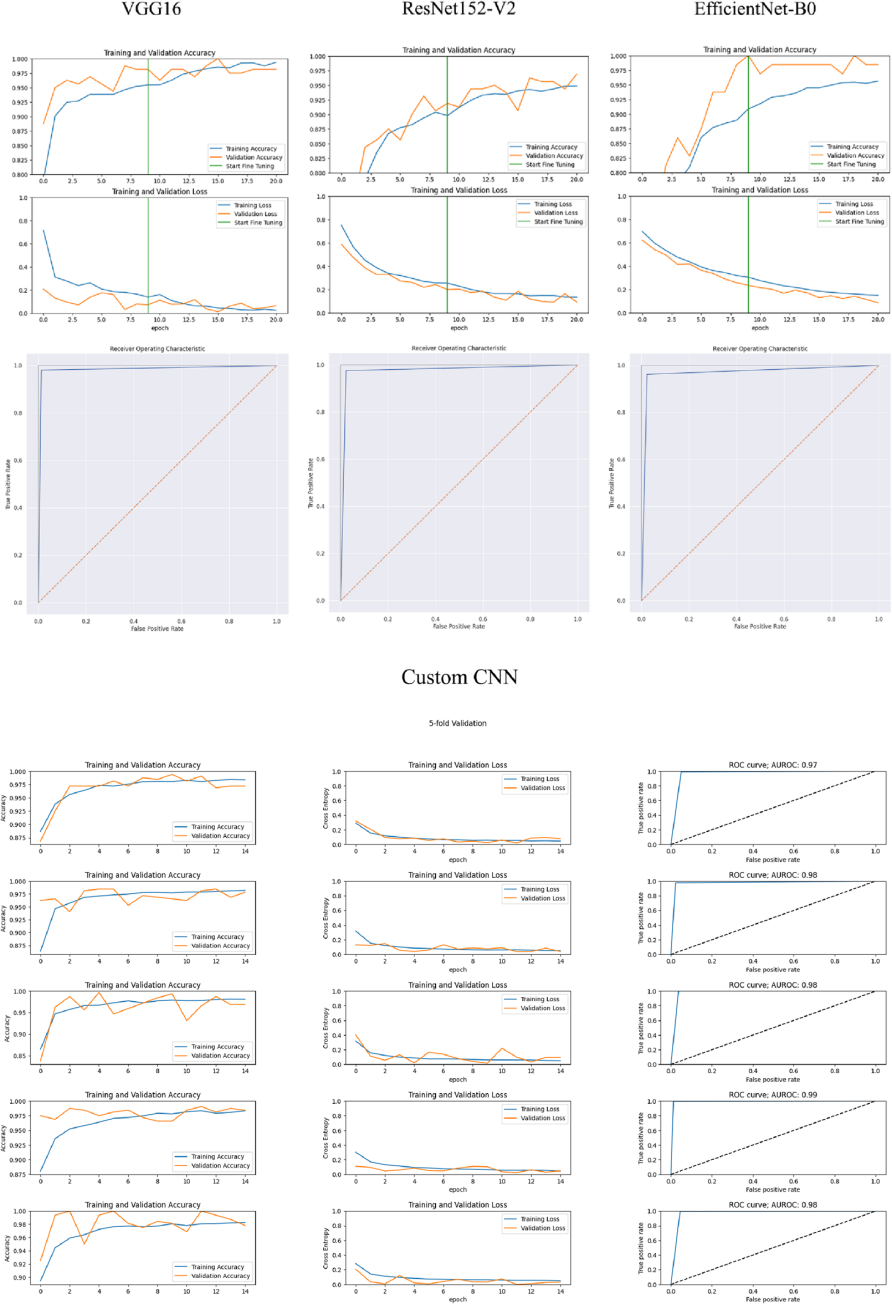
The training results and AUC of the all CNN models. The AUC was 0.99 in VGG16 (*top left*), 0.96 in ResNet152 (*top middle*), 0.96 in EfficientNet-B0 (*top right*), and 0.97 in customized CNN (*bottom*).

We developed CNN models for classifying corneal types in cases for keratoconus and normal before and after generative images with the VAE model. Table 3 demonstrated that the utilization of synthesized images during training process increased classification performance. After training, all of the CNN models showed reasonable accuracy, and no evidence of overfitting was noted when the test dataset was applied (Fig. 5). The accuracy, sensitivity, specificity, PPV, NPV, AUC are shown in Table 3. The highest accuracy level of 0.993 was obtained employing VGG16 model with (sensitivity 0.994, specificity 0.987), followed by ResNet152-V2 (0.959) with (sensitivity 0.959, specificity 0.953), EfficientNet-B0 (0.952) with (sensitivity 0.944, specificity 0.983), and customized CNN (0.974) with (sensitivity 0.980, specificity 0.966). The area under the receiver operator characteristic curve was 0.988 for VGG, 0.964 for ResNet152-V2, 0.963 for EfficientNet-B0, and 0.973 for customized CNN, as illustrated in Fig. 5 (*bottom)*. The performance of each model was deemed acceptable, with VGG16 exhibiting the best performance.

**Table 3:** Results of CNN models before and after generating images with the VAE model.

Models	Data	Accuracy	Sensitivity	Specificity	PPV*	NPV*	AUC
VGG16	Original	0.962	0.959	0.974	0.980	0.949	0.966
	Synthesized	0.993	0.994	0.981	0.994	0.993	0.988
ResNet152-V2	Original	0.939	0.945	0.923	0.928	0.965	0.939
	Synthesized	0.959	0.959	0.953	0.956	0.954	0.964
EfficientNet-B0	Original	0.943	0.953	0.900	0.934	0.948	0.926
	Synthesized	0.952	0.944	0.983	0.971	0.949	0.963
Customized CNN	Original	0.950	0.954	0.960	0.950	0.972	0.957
	Synthesized	0.974	0.980	0.966	0.967	0.970	0.973

*In prevalence of 44%.

**Figure 6:**
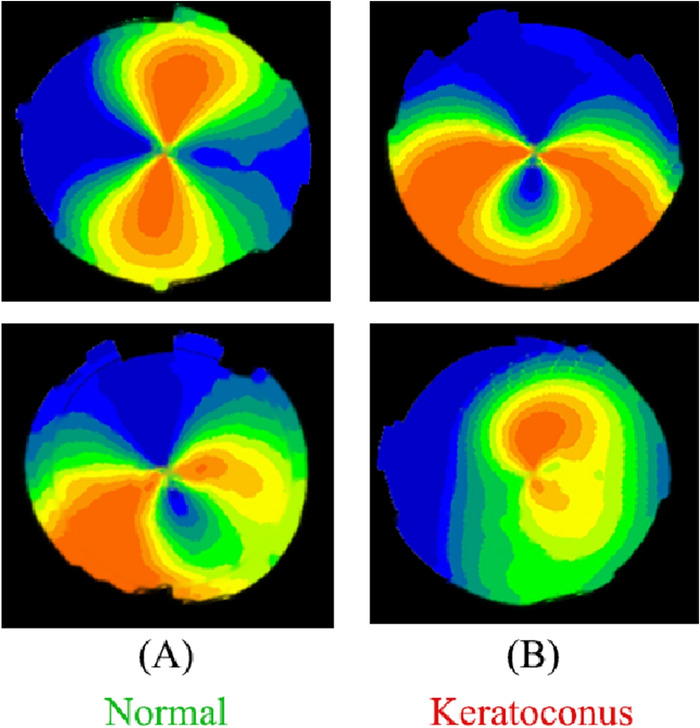
The example of the trained VGG16 model.

The outcomes of DL classification were shown in Fig. 6 using VGG16 model as an example. The normal group consists of (A), flat topographic feature and regular astigmatism, while the keratoconus group consists of (B), steep topographic feature. According to the algorithm, (A) are both normal and predicted as 86% (top) and 89% (bottom) of cases, respectively; (B) are both keratoconus feature and predicted as 98% (top) and 92% (bottom) of cases, respectively. As a result, the present algorithm effectively and correctly distinguished between keratoconus and normal.

**Figure 7:**
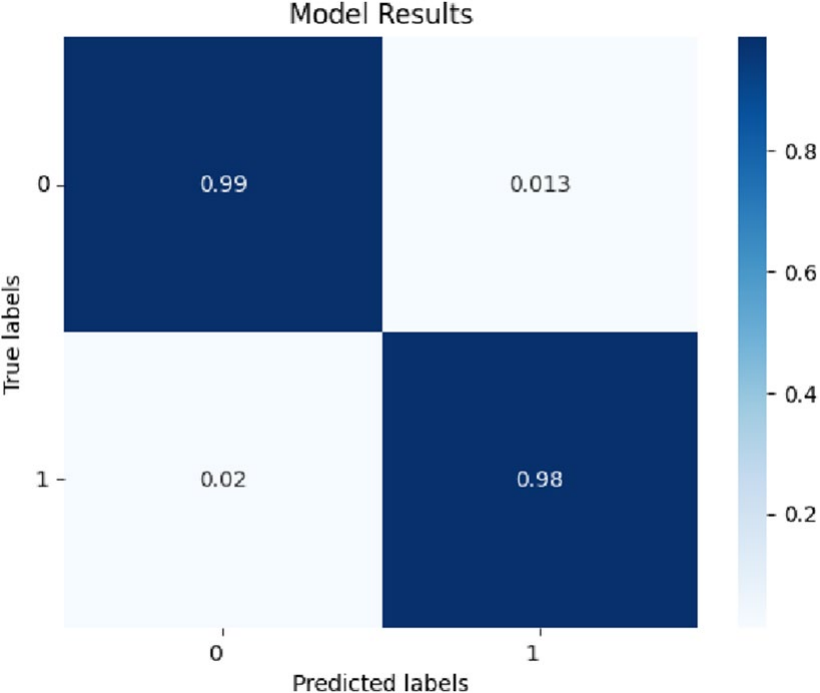
Confusion matrix of VGG 16 model for KCN diagnosis was obtained during evaluation step on test dataset.

**Figure 8:**
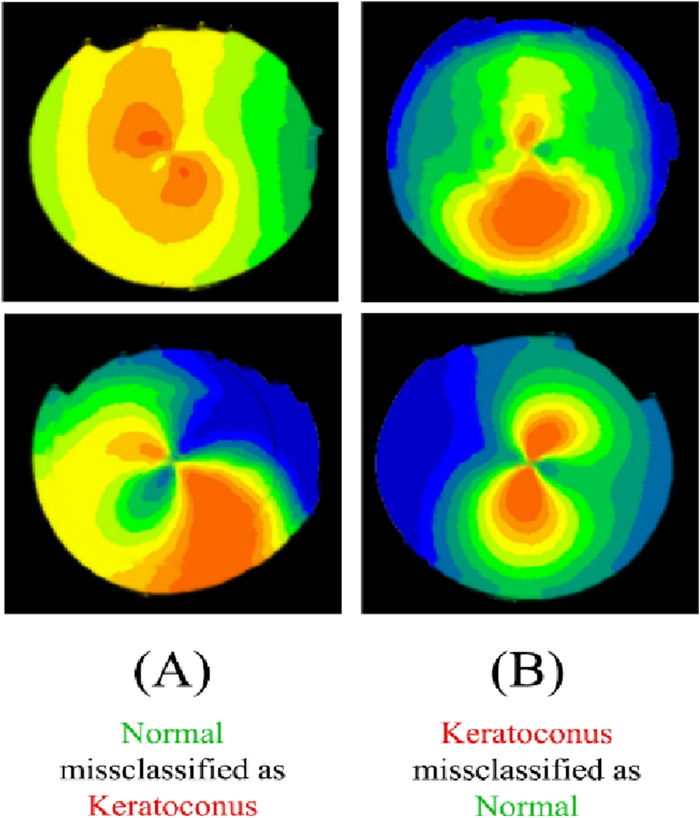
Four sample images that were misclassified by the DL model for VGG16. Two normal eyes that were misclassified as KCN (**A**). Two KCN eyes that were misclassified as normal (**B**).

We additionally calculated the confusion matrix to assess the performance and quality of the learning process. The confusion matrix of VGG16 is presented in Fig. 7. Out of the 800 images, there were only thirteen misclassifications, including five cases of KCN eyes were incorrectly classified as normal. Figure 8 illustrates examples of eyes misclassified by the DL model for VGG16.

**Figure 9:**
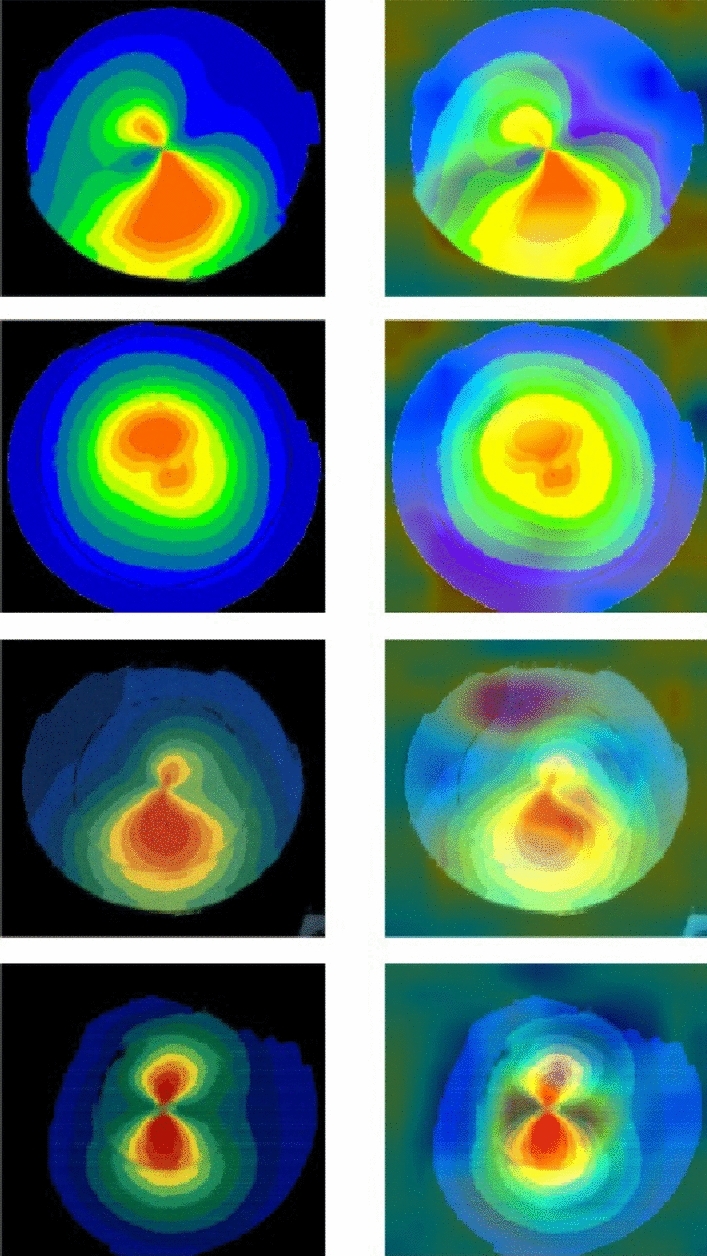
The visualization of the trained CNN models. The left column shows original topographic images. The right column is the heat map visualization as a result of Grad-Cam method which demonstrates the most significant area in the topographic images.

We also included Grad-Cam outputs, a widely accepted approach for visualizing feature maps and pinpointing the most salient regions of interest within the final layer of deep CNN models. The quality of these visualizations was further enhanced through the application of a heat map mixture technique. This refinement contributes to the production of higher-quality figures, which, in turn, serves as a robust means of validating the models and their associated trained parameters (Fig. 9).

## Discussion

We developed multiple DL models to classify keratoconus from non-invasive corneal topography images. In order to solve some of the problems and limitations in previous models, we employed a novel design approach. Large representative datasets are often necessary for DL models to effectively learn various features associated with the underlying condition. In this study, we utilized a relatively large dataset consisting of 4000 corneal images. We employed the VAE model to generate and augment the images. VAEs can be great for feature extraction. The utilization of VAE assists the network in acquiring the ability to generate outputs from a continuous distribution, enabling it to process diverse inputs and produce the desired outcomes^24,25^. It is worth mentioning that one of the capabilities of auto-encoders, as unsupervised learning models, is to cluster data and assign relevant classes as labels. In this study, despite the predetermined labels for the reconstructed data, this aspect was applied as an effective representation of the customized VAE. The VAE model was used to synthesize images from original images, and the resulting images are displayed in Fig. 4. The high quality of these images is evident, and it is also apparent that the structures and morphologies of the images are stable. The study found that the diagnostic accuracy of VGG16, ResNet152-V2, EfficientNet-B0, and customized CNN classifiers improved significantly after using synthetic data samples. Specifically, the diagnostic outcomes for these classifiers increased from 0.962 to 0.993, 0.939 to 0.959, 0.943 to 0.952, and 0.950 to 0.974, respectively. The results from Table 3 indicate that the use of synthetic data samples can enhance the variability of the input dataset, leading to more precise clinical decisions.

Some researchers also have utilized approaches to data augmentation to enhance the training process. These methods involve generating high-quality sample images through the use of a generative model called generative adversarial networks (GANs)^26,27^During a corneal diseases diagnosis task, Hwang et al.^28^ demonstrated that synthetic data augmentation using CGANs improves accuracy by approximately 13% compared to traditional data augmentation methods. The Xception classifier achieved the highest level of performance with 90.5% when using synthesized data. The utilization of conditional GAN for data augmentation has been found to enhance the segmentation accuracy of retinal OCT images, as reported in a previous study^29^. In several studies, GAN has been utilized to increase the amount of data available for analysis in various eye conditions. For instance, it has been used to augment anterior OCT images for angle-closure glaucoma, ocular surface images for conjunctival disease^30^, and corneal topography images for keratoconus detection. They evaluated the performance of the VGG-16 DCNNs to classify a test set using six distinct combinations of both original and synthesized images during the training process. Similar to our study, the VGG16 model obtained the highest accuracy of 99.78%^31^. These findings suggest that incorporating synthetic data samples into the training process of medical image classifiers can improve their diagnostic accuracy and ultimately benefit patients.

Several studies have exclusively employed corneal topography parameters^20,32^. Kmax, I-S and KISA have been utilized as parameters, however there are still challenges with their utilization, including high false-positive rates, complexity, overlap between parameters of normal and KCN eyes, and the number of accessible parameters, which can make interpretation complicated^33^. These studies highly depend on manually created features or machine-extracted indices such as SVM^34^, logistic regression^35^, random forest^36^, decision trees^37^, and neural networks^38^. DL models can offer a complete solution that learns to extract features without supervision, without the need for manually created features or produced parameters^16,19–21^.Since color-coded maps can provide more visual information than topographic and tomographic numeric indices for this learning, we employed the entire images of color-coded maps for deep learning.

In this study, we developed multiple DL models, each trained to extract pertinent deep features from the corneal topographic maps to detect KCN. Our results demonstrated that the utilization of deep learning offered the highest accuracy of 0.99 and AUC 0.99 using the VGG16 model in distinguishing between KCN and normal groups (Table 3). These findings suggest that deep learning can potentially improve the diagnostic precision of keratoconus. All models exhibited a sensitivity and specificity exceeding 0.94. The VGG16 model achieved the highest sensitivity of 0.99, closely followed by the customized CNN with a sensitivity of 0.98. Both the VGG16 and EfficientNet-B0 models demonstrated the highest specificity of 0.98. High sensitivity indicates a low rate of false-negative predictions, which implied that the trained CNN models were appropriate for keratoconus screening. Furthermore, the models exhibit high specificity, indicating strong predictive capability for the normal group. The central cone and the asymmetric bowtie with a skewed radial axis (AB/SRAX) (Fig. 6B (bottom)) are two typical topographic patterns of keratoconus that our algorithms were able to recognize in addition to the inferior steep pattern. Figure 8 presents sample images of eyes that were misclassified by the VGG16 model. Out of 800 samples, there were thirteen misclassifications, including five KCN-positive eyes that were incorrectly labeled as normal. The reason for this issue can be due to the similarity of regular astigmatism pattern in normal cornea with bowtie’s in keratoconus.

Our study demonstrated that the customized CNN model is a promising approach for achieving accurate predictions while minimizing network complexity. Although the VGG16 model outperformed other models in this study, the customized CNN model achieved satisfactory results with an accuracy and AUC of 0.97 at a much faster processing speed compared to other models, which is a strength of our study. This may be due to the ease of training the network to extract suitable features from the data with fewer convolutional layers and relevant filters. Therefore, while deep CNNs are strong in feature learning and obtaining suitable weights, it is possible to achieve similar prediction quality with the customized CNN model with much less network complexity in a more optimal time. Furthermore, the GradCam results, depicted in Fig. 9, demonstrate that the model concentrates its attention on the central area, which is considered the region of clinical significance.

In the study by Abdülhüssein et al.^39^, VGG-16, a pre-trained CNN model, was employed to identify distinct topographic maps. The classification accuracy achieved for SAG, EF, EB, and CT maps was 88.8%, 98.9%, 94.8% and 94.5%, respectively. It is important to mention that the evaluation was conducted on separate training and testing sets, without a validation set. A comparison of previous research into KCN detection^19–23,40^ is provided in Table 4, which also contains information on the device used, the number of eyes, the DL models used, and the evaluation methods.

**Table 4:** The detection of KCN from corneal topographic images in the previous literature.

Study	Groups labeling technique	Corneal imaging modality	Dataset	Evaluation method	Machine learning method/s used	Accuracy
Kamiya et al.^19^	Normal and 4 grades of KCN	Tomey CASIA	543 cases	Fivefold CV	ResNet-18	99%
Kuo et al.^20^	Normal, KCN	Tomey TMS-4 Corneal Topographer	354 cases	Training, testing, and subclinical testing	VGG16	93.1%
					InceptionV3	93.1%
					ResNet152	95.8
Lavric and Valentin^21^	Normal, KCN	Synthetic maps	SyntEyes and SyntEyes KTC models^41^/1 map	Training, validation, and testing	KeratoDetect	99.3%
Zeboulon et al.^22^	Normal/KCN and history of refractive surgery	Bausch + Lomb Orbscan	3000 cases	Tenfold CV	CNN	99.3%
Al-Timemy et al.^23^	Normal, KCN	OCULUS Pentacam	534 cases	Training, validation, and testing	EDTL with AlexNet and product fusion	98.3%
Al-Timemy et al.^40^	Normal, KCN, suspected KCN	OCULUS Pentacam	692 eyes	Training, validation, and independent testing	EfficientNet-B0 DL with SVM	Two-class, 98% Three-class, 81.6%
This study	Normal, KCN	Tomey TMS-4 Corneal Topographer	1758 eyes	Training, validation, and testing	VGG16	99.3%
					ResNet152-V2	95.9%
					EfficientNet-B0	95.2%
					Customized CNN	97.4%

Although we utilized a substantial dataset and a reliable platform, our study has some potential limitations. First, since the data were gathered from two different clinical settings in Mashhad, it is essential to collect data from populations of other races in order to independently evaluate the models and assure generalizability. Second, this study used front topographic corneal maps, which may produce comparable results to other topographic maps from different platforms. However, future research should investigate the impact of fusing different corneal maps and their combinations on the accuracy and generalizability of the results. Third, other corneal disorders, such as subclinical keratoconus was not included in this study due to insufficient availability of relevant images. Further research is recommended to explore the potential of using the GAN model for corneal image synthesis in order to achieve better results and evaluate the differences between corneal maps of normal eyes and eyes with suspected KCN.

## Methods

### Study population

This retrospective study received approval from two crowded tertiary eye clinics, namely Noorafarin and Didar in Mashhad, and was conducted in accordance with the principles outlined in the Declaration of Helsinki. Informed consent was provided by all patients. Initially, we collected 1900 corneal images from overall 1127 subjects were included from September 2015 to June 2021. Most of the patients were candidates for refractive surgery. Subjects with the previous ocular surgery, trauma, corneal degenerations, and contact lens discontinuation less than three weeks had been excluded. Ultimately, a total of 1010 subjects with 1758 images were included in this study. The sample sizes were as follows: 978 images of normal corneas from 535 subjects and 780 images of KCN from 475 patients. The medical records of each patient had been reviewed and retrieved to confirm the diagnosis of KCN. Each patient’s records consisted of the results of optometry and ophthalmology examinations including slit lamp biomicroscopy, dry and cycloplegic refraction, and uncorrected and best-corrected visual acuity. To diagnose at-risk corneas, Corneal topography, tomography, and biomechanical corneal characteristics were evaluated using Tomey (TMS-4N, Tomey Corp.), Pentacam HR (Oculus, Wetzlar, Germany), and Corvis ST (Oculus, Wetzlar, Germany) devices respectively. There are three corneal specialists were involved in the assessment, diagnosis and labeling of the keratoconus. The initial corneal topographic maps were collected with the use of (TMS-4N; Tomey Corporation, Nagoya, Japan).

### Data preprocessing and algorithms

**Figure 10:**
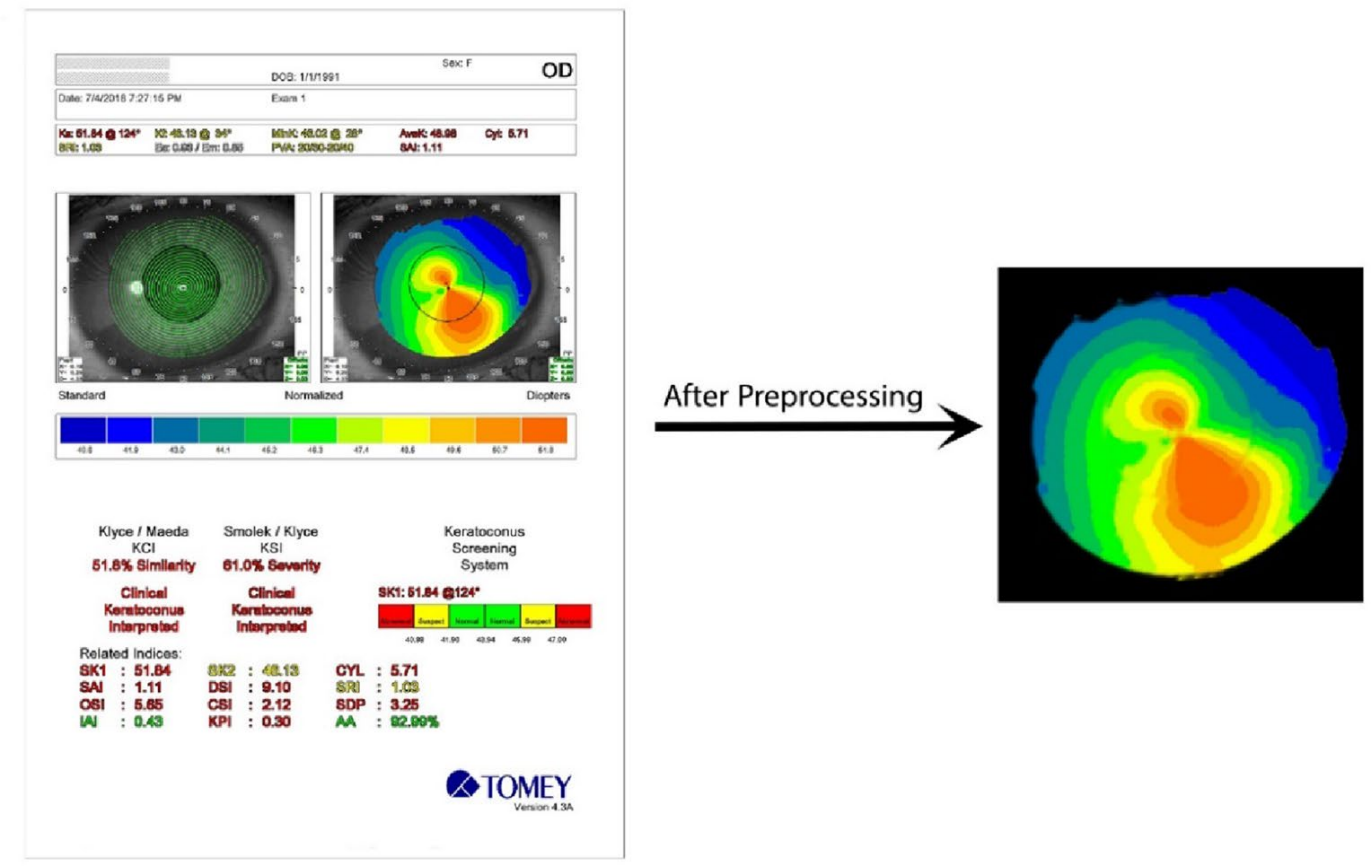
A raw sample data with regular noise.

At first, data preprocessing was applied to eliminate irrelevant elements from the images, such as words and numbers. To achieve this, we utilized computer vision algorithms to crop and extract the cornea pattern in the images. Then a HSV mask was applied to filter out the segment of the cornea and the extra margins were removed, so the cornea was obtained. In the next step of the preprocessing, it was observed that a significant number of images acquired from high-resolution medical imaging devices, such as the TMS-4, were contaminated with regular noise. As a solution, a noise removal function was designed to denoise the images. This process was applied individually to all images. Eventually, a collection of high-quality images with normalized sizes were obtained for training deep learning models. Figure 10 shows a raw sample data with regular noise and its result after preprocessing.

### Variational autoencoder (VAE) to augment images

One of the major challenges faced in the medical field is the scarcity of large-scale datasets. In this study, we proposed an innovative approach to address this issue by utilizing Variational Auto-Encoders (VAEs) to generate and augment images. Auto encoders are a combination of statistics and information theory, combined with the power of deep neural networks. They are efficient at solving generative problems for high dimensional data.

Variational auto encoders, in particular, focus on understanding the latent representation of data and provide a way to generate new samples using a probabilistic approach^42^. The VAEs is a deep neural network that utilizes unsupervised learning. It is composed of two main components: an encoder and a decoder network, which are separated by a layer known as the latent variable layer or latent space. VAEs are often employed as generative models because they are able to extract useful features and learn a suitable representation of the data through the encoder, and then generate output that is in the same format as the original data by using the decoder which takes the latent representation as input^24,43^. The encoder component of a VAE, when presented with an image input, produces a two-parameter latent vector representation through a sequence of down-sampling operations, such as convolutions. Similarly, the decoder component, when given a one-parameter latent vector representation, reconstructs the original input data via a series of up-sampling operations, such as transposed convolutions^25^.

In our research, we employed a VAE model that comprises of both encoder and decoder networks, which are deep convolutional neural networks with 3 Convolutional layers and 1 fully connected layer in each. In addition to the input layer with a shape of 104 × 104 × 1 in dimensions, respectively, the architecture of the encoder network is 64-32-16-128, where 64, 32 and 16 represents the number of filters in the convolutional layers while 128 is the number of hidden neurons in the fully connected dense layer. The architecture of the decoder network is 2704-16-32-64, where 2704 is the number of hidden neurons in the deep net. After applying convolutional layers and performing down-sampling in the encoding process, a feature vector of size (13 × 13 × 16) was obtained by the flatten layer and passed to a dense layer with 128 input neurons and 2 output parameters which are mean and standard deviation of the data distribution. These two parameters are delivered to the latent space and a single sampling variable is provided by the latent layer and passed to the decoder model as an input sample.

**Figure 11:**
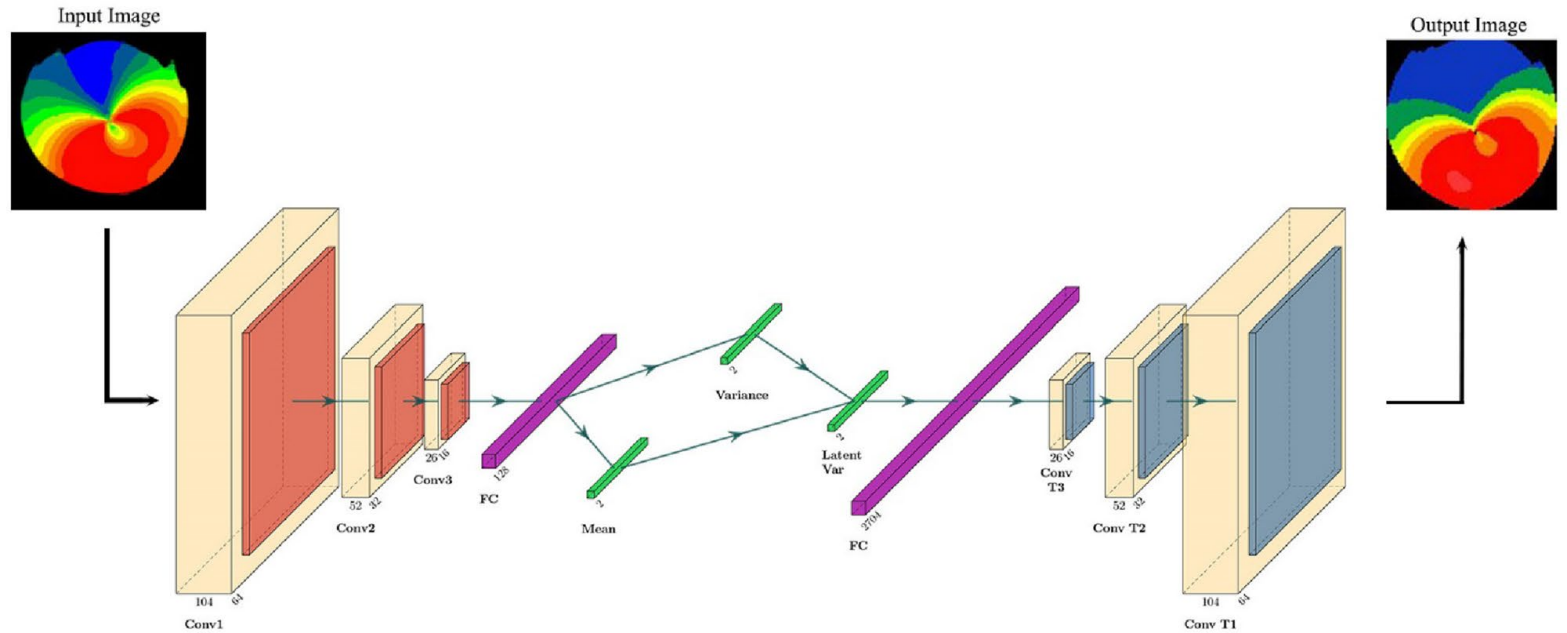
Architecture of the VAE in the case study. As shown in the figure, a preprocessed sample is first converted to grayscale and then fed to the network. Final result is decoded back to the original shape and color space.

Having order of the mentioned encoding procedure reversed, the decoder model takes a single sampling variable vector as its input which is passed to a dense layer with the same number of neurons equal to the number of extracted features by the encoder, followed by a reshape layer. The resulting feature vector is then subjected to a series of up-sampling steps using three consecutive transposed convolutional layers, resulting in a final output vector of the original size of the input sample. Figure 11 shows the VAE architecture that was developed to generate images from corneal topographics.

The loss function of our VAE model like most of variational auto-encoders is based on 2 loss functions namely reconstruction loss and Kullback–Leibler (KL) divergence. The reconstruction error is an indication of the quality of the generated samples. The lower the error, the more optimized the generative performance. KL loss, however, aims to measure the divergence distance and dissimilarity of two distributions based on information theory and is used as a regularization technique for the latent space^44^.

VAEs are interpreted as Bayesian inference model, where the prior distribution of the latent variable z is represented by *p(z)*. The generative model for an observation x is defined as *p(z|x)* and the inference model for the latent representation of the data is defined as *q(z|x)*. The objective of the VAE loss function is to minimize the KL divergence distance between the prior distribution *p(z|x)* and the inferred distribution *q(z|x)*.

$$\mathit{loss}= \mathit{min}KL \left(q\left(z|x\right)\mid \mid p\left(z|x\right)\right).$$

Instead of trying to minimize the KL divergence of the above term, we can simplify the loss function by re-arranging it as a maximization objective using the decoder output *y* for the input data *x* as follow:

$$loss= {E}_{q\left(z|y\right)}\mathit{log}p\left(y|z\right)- KL\left(q\left(z|x\right)\mid \mid p(z)\right).$$

The first term in the above equation describes the log-likelihood of the reconstruction, while the second term represents an attempt to make the learned distribution *q* and the true prior distribution *p* as similar as possible by minimizing their distance^45^. Hence, the total loss function of the VAE model for *N* input data of encoder ${\{{x}_{i}\}}_{i=1}^{N}$ and *N* output samples of decoder ${\{{y}_{i}\}}_{i=1}^{N}$ with latent variable *z* can be shown below:

$$loss=\sum_{i=1}^{N}\left\{{ E}_{q\left(z|{y}_{i}\right)}\mathit{log}p\left({y}_{i}|z\right)- KL \left[q\left(z|{x}_{i}\right)\mid \mid p\left(z\right)\right] \right\}.$$

**Figure 12:**
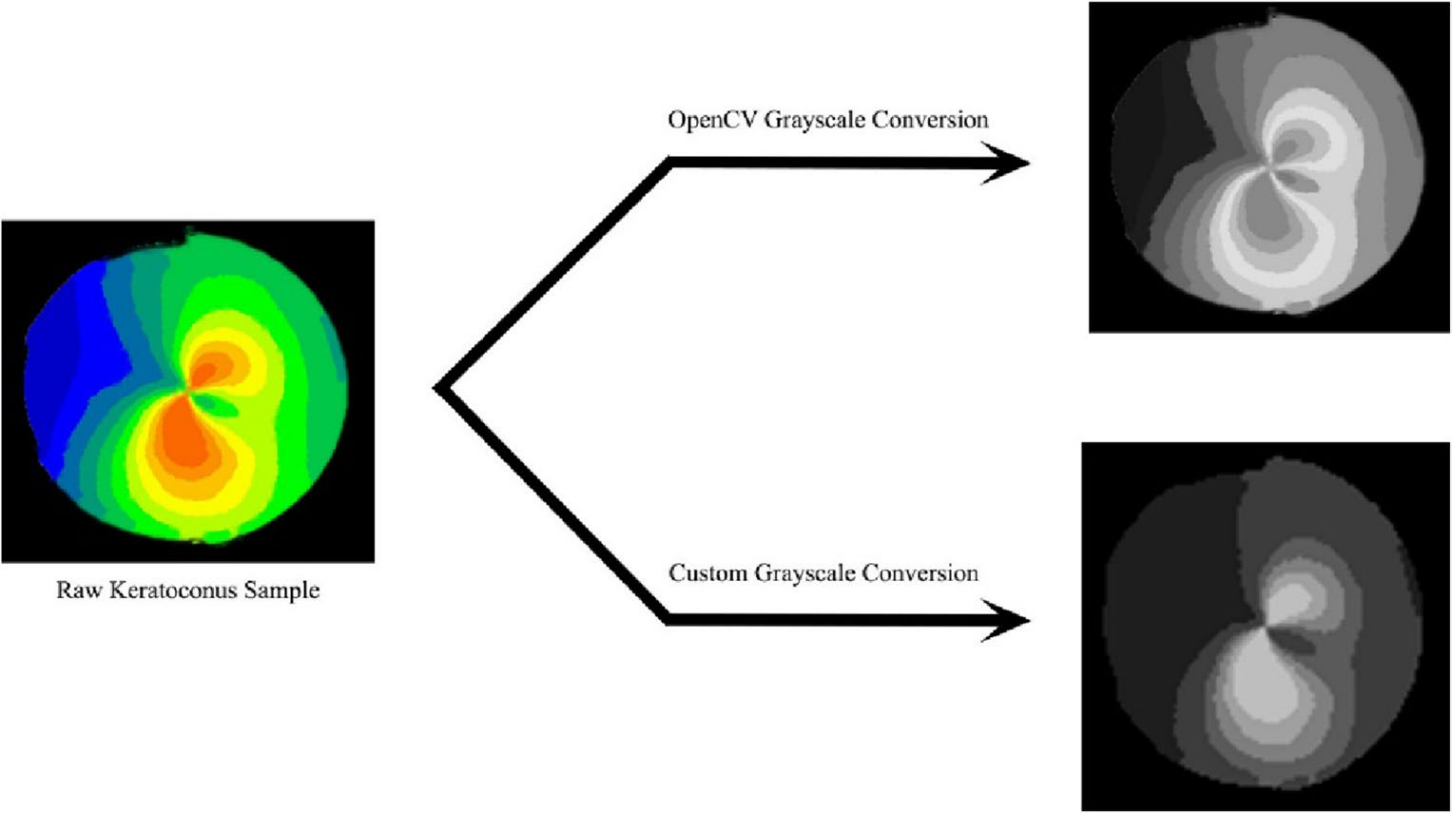
Comparison of grayscale conversion results between OpenCV and the manually implemented methods.

Before training our VAE model, it is necessary to prepare the raw dataset by converting all images to grayscale and adjusting their resolution. This is because unsupervised generative models tend to perform better when working with single channel images that are preprocessed in this way. A custom preprocessing method was designed to convert all images to grayscale while preserving the significance of color spectrum, borders and other features in the images. This is because, later in the classification task, one of the key features that distinguishes keratoconus patients from normal ones is the organization of the cornea color in the clinical images. Therefore, standard predefined methods such as the OpenCV grayscale conversion method could not be used, as they would not maintain the important features of the images. Figure 12 illustrates the difference of both conversions.

For the purpose of training, we used random horizontal flip and rotation of 10% or less to augment the training dataset. With a learning rate of 0.0001 and RMSprop optimizer, the training task was performed for 50 epochs across 9 distinct versions of our VAE models beginning with the initial model characterized by relatively lower implementation complexity and extending to the latest versions distinguished by enhanced code efficiency and optimized model parameters. As we trained our most accurate VAE model on the raw dataset and constructed new appropriate images, the topographic pictures were divided into the subsequent of two datasets: (a) training and (b) test datasets. We used 3200 images [%80] (1600 keratoconus and 1600 normal) in the training dataset, and 800 images [%20] (400 keratoconus and 400 normal) in the test dataset. We allocated 10% of the training dataset to validation dataset. Unlike the training dataset, which comprised a combination of both generated and original images, the test dataset exclusively consisted of original images. This approach was adopted to ensure the accuracy and reliability of model metric measurements. The test dataset did not involve the training process.

### Deep learning architectures and visualization

In this study, we presented four methods for classifying patients with keratoconus from normal samples, using convolutional neural network (CNN) architecture. Three of these methods are based on transfer learning and fine-tuning of pretrained models including VGG16^46^, EfficientNet-B0^47^, and ResNet152^48^ models on a custom dataset. The fourth method is a bespoke CNN model implemented from scratch^49,50^.

The VGG16 model is a 13-layer convolutional neural network (CNN) composed of 5 max-pooling layers and 3 fully connected layers. It is characterized by the use of max-pooling layers every 2 or 3 convolutions, with an increase in the number of 3 × 3 filters from 64 in the first convolutional layer to 512 in the last. The final prediction of the model is made by the SoftMax classifying layer stacked on top of the flattened and fully connected dense layers^51,52^.

The EfficientNet-B0 model is the foundation of the EfficientNet family and utilizes a CNN architecture with the aim of uniformly scaling all dimensions of depth, width, and resolution. The compound scaling method balances the need for additional layers to increase the receptive field and channels to capture more detailed patterns on larger images. The base model is constructed from Mobile Inverted Bottleneck conv (MBConv) blocks from MobileNetV2, along with squeeze-and-excitation blocks^53^.

Deep residual network, similarly, utilizes a combination of convolutional, pooling, activation and fully-connected layers. It differs from other networks due to its identity connections between residual blocks, which helps prevent the vanishing gradient problem in the backpropagation process. This model comprises of bottleneck design, with each block consisting of 1 × 1, 3 × 3 and 1 × 1 convolutional layers. The network concludes with an average pooling layer and a fully-connected layer with a single neuron, producing a binary classification output^54^.

**Figure 13:**
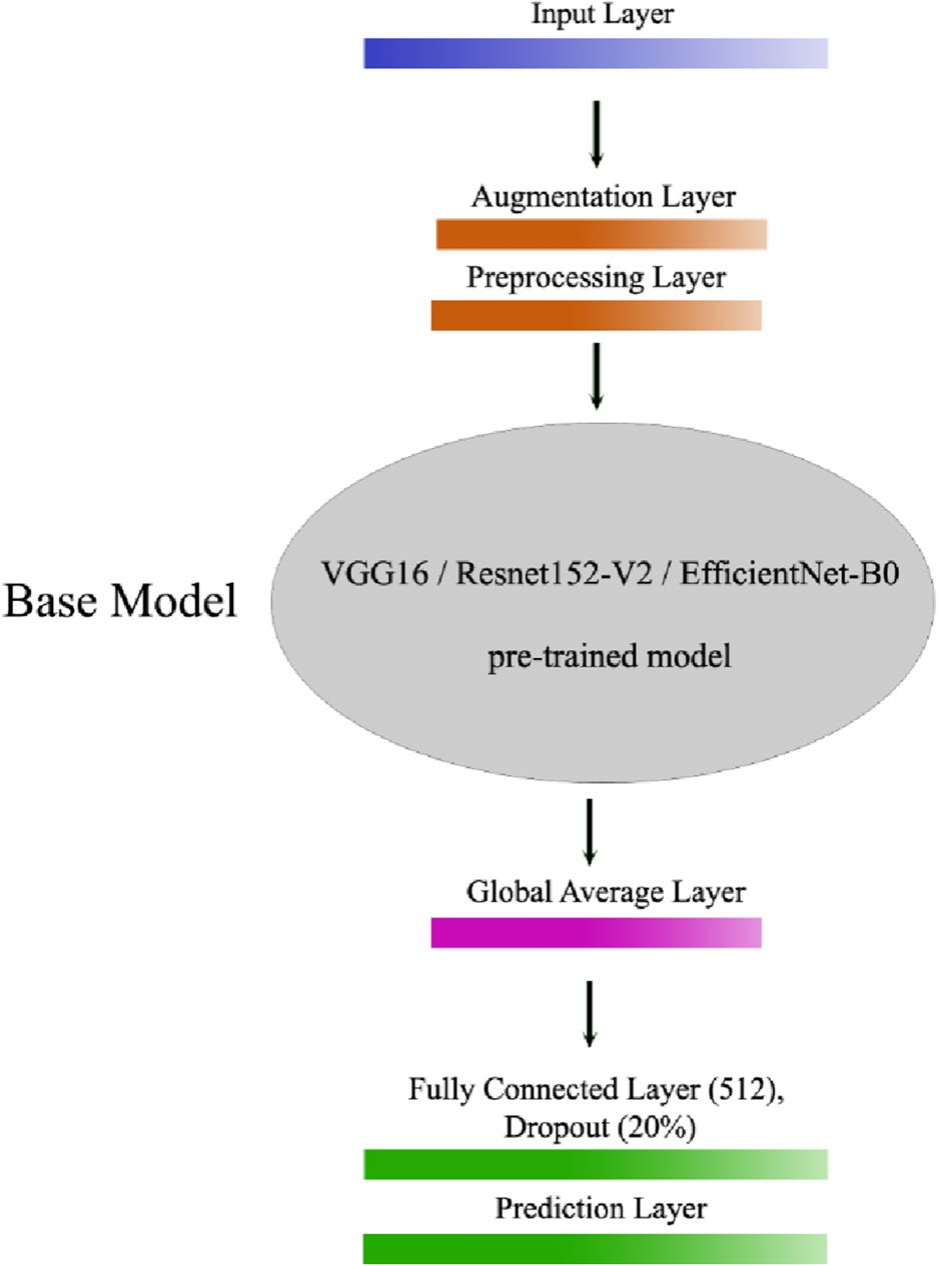
Architecture of the present CNNs for keratoconus.

The three CNNs were implemented with the pre-trained weights from the ImageNet dataset. The shapes of their input layer were in the order of 224 × 224 × 3, 160 × 160 × 3 and 224 × 224 × 3. To augment the performance of the networks, a data augmentation layer was added, consisting of a random horizontal flip and 20% random rotation. This was followed by a preprocessing layer, which rescaled the pixels between 0 and 255 to the range of [− 1, 1]. The CNNs were then linked to the previous layers and a classification head was added on top, including a global average pooling layer followed by a dense layer with 512 neurons and a dropout rate of 0.2. Finally, a single neuron prediction layer was added to make the final predictions. Figure 13 demonstrates the mentioned structures.

The study also utilized a customized CNN architecture that was designed to process 3-channeled images with a size of 50 × 50. This network comprised of three convolutional layers with 64, 32, and 16 3 × 3 filters respectively and a stride of 2. The convolutional layers were connected to two fully connected layers, with a dropout layer of 0.25 rate placed in between. The final prediction was made by a single neuron layer with a sigmoid activation function.

In the training of pretrained networks, the common approach is to utilize the features learned by a model that trained on a larger dataset in the same domain when dealing with a small dataset. This is achieved by instantiating the pre-trained models and appending a fully-connected classifier. The pre-trained models are fixed, and only the weights of the classifier are updated during training. In this scenario, the convolutional base extracted all of the features related to each image and a classifier was trained to determine the image class based on the extracted features. Consequently, the models were trained and validated for 15 epochs using a learning rate of 0.0001, the Adam optimizer, and the binary cross-entropy loss function, with all layers of the base CNN model being kept in a frozen state. In the feature extraction experiment, only the top layers of the pre-trained networks were trained while keeping the base model's weights unchanged. To further improve performance, the top layers of the pre-trained models were fine-tuned by making the same number of convolutional layers trainable in all CNN models. The fine-tuning process was carried out by retraining the whole networks for an additional 10 epochs, forcing the weights to be tuned from generic feature maps to features associated specifically with the dataset^55^.

The customized CNN model was trained using a fivefold cross-validation approach with random shuffling of the dataset, and each fold was trained for 15 epochs. The learning rate and loss function were consistent with those used for the pre-trained CNN models, but the RMSprop optimization algorithm was utilized in place of the Adam optimizer.

### Computer hardware and software

The deep learning computations described in this study were executed on a personal computer equipped with an AMD Ryzen core 5 3600 processor at 3.59 GHz and an NVIDIA GeForce GTX 1650 GPU. The deep neural network was developed using the Python programming language, utilizing the TensorFlow 2.3.0 and Keras 2.4.3 libraries.

### Statistical analysis

For demographic data, a chi-square test was employed to compare gender distribution between keratoconus and normal groups, while a t-test was used to assess differences in age. The performance of our DL algorithm were evaluated based on measures such as area under the receiver operating characteristic curve (AUC), confusion matrix, accuracy, sensitivity, specificity, positive predictive values (PPV), and negative predictive values (NPV). The ROC curves were utilized to specify the overall predictive accuracy of the examined parameters, indicated by AUC, and to calculate the specificity and sensitivity in distinguishing KCN from normal eyes. The Optimal cutoff points for each index were received from the ROC curves, selecting the points closest to the maximum value of sensitivity equals specificity^19^. All statistical analyses were achieved using SPSS software (SPSS 24.0; SPSS Inc., Chicago, IL, USA) and a P-value less than 0.05 was considered statistically significant. Additional metrics were obtained using the Scikit-learn and TensorFlow platforms.

## Conclusions

The study utilized a relatively large dataset consisting of 4000 images with the VAE approach to construct various CNN models for extracting deep features from corneal topographic maps. The results demonstrate the effectiveness of transfer learning in generating efficient deep classifiers, leading to highly accurate models in distinguishing between KCN and normal eyes. We demonstrated that the utilization of synthesized images during training process increased classification performance. The implementation of the automated keratoconus model shows great potential for enhancing clinical practices, aiding corneal specialists in the identification and management of KCN patients, and contributing to a reduction in the number of corneal transplant cases.

## Data Availability

Data will be made available on reasonable request from the corresponding author.

## References

[1] Sharif R, Bak-Nielsen S, Hjortdal J, Karamichos D (2018). Pathogenesis of Keratoconus: The intriguing therapeutic potential of Prolactin-inducible protein. Prog. Retin. Eye Res..

[2] Kelly T-L, Williams KA, Coster DJ (2011). Corneal transplantation for keratoconus: A registry study. Arch. Ophthalmol..

[3] Buzzonetti L, Bohringer D, Liskova P, Lang S, Valente P (2020). Keratoconus in children: A literature review. Cornea.

[4] Georgiou T, Funnell C, Cassels-Brown A, O'conor R (2004). Influence of ethnic origin on the incidence of keratoconus and associated atopic disease in Asians and white patients. Eye.

[5] Rafati S (2019). Demographic profile, clinical, and topographic characteristics of keratoconus patients attending at a tertiary eye center. J. Curr. Ophthalmol..

[6] Galvis V (2015). Keratoconus: An inflammatory disorder?. Eye.

[7] Tsai, Y.-Y., Chen, P.-Y. & Ho, T.-Y. In *International Conference on Machine Learning*, 9614–9624 (PMLR, 2020).

[8] You A, Kim JK, Ryu IH, Yoo TK (2022). Application of generative adversarial networks (GAN) for ophthalmology image domains: A survey. Eye Vis..

[9] Tong Y, Lu W, Yu Y, Shen Y (2020). Application of machine learning in ophthalmic imaging modalities. Eye Vis..

[10] Smolek MK, Klyce SD (1997). Current keratoconus detection methods compared with a neural network approach. Investig. Ophthalmol. Vis. Sci..

[11] Arbelaez MC, Versaci F, Vestri G, Barboni P, Savini G (2012). Use of a support vector machine for keratoconus and subclinical keratoconus detection by topographic and tomographic data. Ophthalmology.

[12] Smadja D (2013). Detection of subclinical keratoconus using an automated decision tree classification. Am. J. Ophthalmol..

[13] Hidalgo IR (2017). Validation of an objective keratoconus detection system implemented in a Scheimpflug tomographer and comparison with other methods. Cornea.

[14] Issarti I (2019). Computer aided diagnosis for suspect keratoconus detection. Comput. Biol. Med..

[15] LeCun Y, Bengio Y, Hinton G (2015). Deep learning. Nature.

[16] Dos Santos VA (2019). CorneaNet: Fast segmentation of cornea OCT scans of healthy and keratoconic eyes using deep learning. Biomed. Opt. Express.

[17] Rawat W, Wang Z (2017). Deep convolutional neural networks for image classification: A comprehensive review. Neural Comput..

[18] Goodfellow I, Bengio Y, Courville A (2016). Deep Learning.

[19] Kamiya K (2019). Keratoconus detection using deep learning of colour-coded maps with anterior segment optical coherence tomography: A diagnostic accuracy study. BMJ Open.

[20] Kuo B-I (2020). Keratoconus screening based on deep learning approach of corneal topography. Transl. Vis. Sci. Technol..

[21] Lavric A, Valentin P (2019). KeratoDetect: Keratoconus detection algorithm using convolutional neural networks. Comput. Intell. Neurosci..

[22] Zéboulon P, Debellemanière G, Bouvet M, Gatinel D (2020). Corneal topography raw data classification using a convolutional neural network. Am. J. Ophthalmol..

[23] Al-Timemy AH, Ghaeb NH, Mosa ZM, Escudero J (2022). Deep transfer learning for improved detection of keratoconus using corneal topographic maps. Cogn. Comput..

[24] Zhao, S., Song, J. & Ermon, S. In *Proc. of the AAAI Conference on Artificial Intelligence*, 5885–5892 (2019).

[25] Cemgil T, Ghaisas S, Dvijotham K, Gowal S, Kohli P (2020). The autoencoding variational autoencoder. Adv. Neural Inf. Process. Syst..

[26] Goodfellow I (2014). Generative adversarial nets. Adv. Neural Inf. Process. Syst..

[27] Yi X, Walia E, Babyn P (2019). Generative adversarial network in medical imaging: A review. Med. Image Analy..

[28] Jameel SK (2022). Exploiting the generative adversarial network approach to create a synthetic topography corneal image. Biomolecules.

[29] Kugelman J (2021). Data augmentation for patch-based OCT chorio-retinal segmentation using generative adversarial networks. Neural Comput. Appl..

[30] Yoo TK, Choi JY, Kim HK, Ryu IH, Kim JK (2021). Adopting low-shot deep learning for the detection of conjunctival melanoma using ocular surface images. Comput. Methods Prog. Biomed..

[31] Abdelmotaal H, Abdou AA, Omar AF, El-Sebaity DM, Abdelazeem K (2021). Pix2pix conditional generative adversarial networks for scheimpflug camera color-coded corneal tomography image generation. Transl. Vis. Sci. Technol..

[32] Kojima T (2020). Keratoconus screening using values derived from auto-keratometer measurements: A multicenter study. Am. J. Ophthalmol..

[33] Maeda N, Klyce SD, Smolek MK (1995). Neural network classification of corneal topography. Preliminary demonstration. Investig. Ophthalmol. Vis. Sci..

[34] Hidalgo IR (2016). Evaluation of a machine-learning classifier for keratoconus detection based on Scheimpflug tomography. Cornea.

[35] Velázquez-Blázquez JS, Bolarín JM, Cavas-Martínez F, Alió JL (2020). EMKLAS: A new automatic scoring system for early and mild keratoconus detection. Transl. Vis. Sci. Technol..

[36] Chandapura R (2019). Bowman’s topography for improved detection of early Ectasia. J. Biophotonics.

[37] Mosa ZM, Ghaeb NH, Ali AH (2019). Detecting keratoconus by using SVM and decision tree classifiers with the aid of image processing. Baghdad Sci. J..

[38] Shi C (2020). Machine learning helps improve diagnostic ability of subclinical keratoconus using Scheimpflug and OCT imaging modalities. Eye Vis..

[39] Abdülhüssein, N. S. *Building smart algorithm to extract features of topographic images of a human eye*, Aksaray Üniversitesi Fen Bilimleri Enstitüsü (2018).

[40] Al-Timemy AH (2021). A hybrid deep learning construct for detecting keratoconus from corneal maps. Transl. Vis. Sci. Technol..

[41] Rozema JJ (2017). SyntEyes KTC: Higher order statistical eye model for developing keratoconus. Ophthalmic Physiol. Opt..

[42] Kingma, D. P. & Welling, M. Auto-encoding variational bayes. Preprint at https://arXiv.org/arXiv:1312.6114 (2013).

[43] Hallett, N. et al*.* In *International Joint Conference on Neural Networks (IJCNN)*, 1–7 (IEEE, 2020).

[44] Asperti A, Trentin M (2020). Balancing reconstruction error and Kullback-Leibler divergence in variational autoencoders. IEEE Access.

[45] Dilokthanakul, N. et al. Deep unsupervised clustering with gaussian mixture variational autoencoders. Preprint at https://arXiv.org/arXiv:1611.02648 (2016).

[46] Simonyan, K. & Zisserman, A. Very deep convolutional networks for large-scale image recognition. Preprint at https://arXiv.org/arXiv:1409.1556 (2014).

[47] Tan, M. & Le, Q. In *International Conference on Machine Learning*, 6105–6114 (PMLR, 2019).

[48] He, K., Zhang, X., Ren, S. & Sun, J. In *Proc. of the IEEE conference on computer vision and pattern recognition*, 770–778 (2016).

[49] Kim I, Rajaraman S, Antani S (2019). Visual interpretation of convolutional neural network predictions in classifying medical image modalities. Diagnostics.

[50] Ting DSW (2019). Artificial intelligence and deep learning in ophthalmology. Br. J. Ophthalmol..

[51] Chollet, F. In *Proc. of the IEEE Conference on Computer Vision and Pattern Recognition*, 1251–1258 (2017).

[52] Howard, A. et al. In *Proc. of the IEEE/CVF International Conference on Computer Vision*, 1314–1324 (2019).

[53] Pham, H., Guan, M., Zoph, B., Le, Q. & Dean, J. In *International Conference on Machine Learning*, 4095–4104 (2022) (PMLR).

[54] Tan, M. & Le, Q. V. Mixconv: Mixed depthwise convolutional kernels. Preprint at https://arXiv.org/arXiv:1907.09595 (2019).

[55] Sharif Razavian, A., Azizpour, H., Sullivan, J. & Carlsson, S. In *Proc. of the IEEE Conference on Computer Vision and Pattern Recognition Workshops*, 806–813 (2014).

