# Author Correction: Computer-aided diagnosis of keratoconus through VAE-augmented images using deep learning

**DOI:** 10.1038/s41598-023-50046-y

**Published:** 2023-12-21

**Authors:** Zhila Agharezaei, Reza Firouzi, Samira Hassanzadeh, Siamak Zarei‑Ghanavati, Kambiz Bahaadinbeigy, Amin Golabpour, Reyhaneh Akbarzadeh, Laleh Agharezaei, Mohamad Amin Bakhshali, Mohammad Reza Sedaghat, Saeid Eslami

**Affiliations:** 1 Pharmaceutical Research Center, Pharmaceutical Technology Institute, Mashhad University of Medical Sciences, Mashhad, Iran; 2 Department of Medical Informatics, Faculty of Medicine, Mashhad University of Medical Sciences, Mashhad, Iran; 3 Department of Computer Engineering, Ferdowsi University of Mashhad, Mashhad, Iran; 4 School of Paramedical Sciences and Rehabilitation, Mashhad University of Medical Sciences, Mashhad, Iran; 5 Eye Research Center, Mashhad University of Medical Sciences, Mashhad, Iran; 6 Medical Informatics Research Center, Institute for Future Studies in Health, Kerman University of Medical Sciences, Kerman, Iran; 7 School of Medicine, Shahroud University of Medical Sciences, Shahroud, Iran; 8 Department of Optometry, School of Paramedical Sciences, Mashhad University of Medical Sciences, Mashhad, Iran; 9 Modeling in Health Research Center, Institute for Future Studies in Health, Kerman University of Medical Sciences, Kerman, Iran

Correction to: *Scientific Reports* 10.1038/s41598-023-46903-5, published online 23 November 2023

The original version of this Article contained an error in the spelling of the author Samira Hassanzadeh, which was incorrectly given as Samira Hasanzadeh.

The original Article has been corrected.

